# Serogroup A *Neisseria meningitidis* outside Meningitis Belt in Southwest Cameroon

**DOI:** 10.3201/eid0910.030170

**Published:** 2003-10

**Authors:** Patrick Cunin, Marie-Christine Fonkoua, Basile Kollo, B. Atembeh Bedifeh, Paul Bayanak, Paul M.V. Martin

**Affiliations:** * Centre Pasteur du Cameroun, Yaoundé, Cameroun; † Direction Générale de la Santé, Yaoundé, Cameroun

**To the Editor:** Epidemic meningitis associated with serogroup A *Neisseria meningitidis* is a devastating disease in the absence of vaccination (*1*). Without treatment, the case-fatality rate is high, approaching 100%. In Africa, such epidemics occur regularly (*1*) within a well-limited geographic zone, the so-called African meningitis belt (*2*). In the countries within the meningitis belt, the illness is endemic and sporadic: numerous cases of meningococcal meningitis are reported each year during the dry season, and every 6–12 years a large outbreak occurs. Serogroup A *N. meningitidis* also causes sporadic cases of meningitis outside the meningitis belt, accounting for 10% to 30% of cases with identified causes (*1*,*3*). Outbreaks may also occur outside of the belt, but they do not exhibit the same epidemiologic aspects. We report an epidemic of meningococcal meningitis in the South-West Province of Cameroon (~500 km south of the African meningitis belt and 350 km east of Yaoundé, the country’s capital), involving 61 cases and 13 (21%) deaths.

Clinical and epidemiologic information was collected from medical records at Bechati Health Centre and Fontem Missionary Hospital, the only two care centers in the epidemic area. A case was defined as sudden fever >38°C and neck stiffness if the patient was >12 months of age, or bulging fontanelle if the patient was <12 months of age. Other symptoms of meningitis, such as nausea, vomiting, irritability, confusion, and lethargy to the point of coma, were observed in several patients. An epidemic threshold of 15 cases reported in a 2-week period in a population of >100,000 has the specificity and probability to predict a meningococcal epidemic within the meningitis belt (*1*). In the Bechati area (10,326 inhabitants), nine fatal cases occurred during the first week (March 5–12, 2000), for an attack rate of 87 per 100,000, a figure that local health authorities considered as epidemic.

The outbreak extended from March 6 to April 6, 2000, and peaked March 21–22 (10 cases in 48 hours), in the area of Bechati (5°40′ north of the equator, 300 km^2^). The first case-patient was retrospectively identified as a 9-month-old child from Bechati, treated for meningitis at Fontem Missionary Hospital on February 25. This child was shown to have a gram-negative *Diplococcus* infection by microscopic analysis of a cerebrospinal fluid (CSF) sample. (Fontem is a rural city, 15 km from Bechati, by a poor-quality railroad track.) The epidemic began on March 5 in Bechati and spread to seven other villages. The last case was recorded on April 6. After the index case, 6 cases occurred in week 1; 16 cases in weeks 2, 3, and 4; and 6 cases in week 5. A total of 61 cases were registered in the Bechati Health District (33 male and 28 female patients), with a mean age of 22 years (range 9 months to 70 years) and an attack rate of 591 per 100,000 (61/10,326).

The first patient was cured by appropriate treatment with thiamphenicol at Fontem Missionary Hospital. The next nine case-patients in these remote villages all died, either without treatment or despite traditional treatment. As this meningococcal epidemic was the first of its kind in this area, introducing an efficient response took some time. The public health authorities introduced chloramphenicol treatment on March 13; subsequently, four more deaths occurred, including two untreated patients, among the next 51 case-patients. The death rate was 100% in week 1 (6/6), 31.3% in week 2 (5/16), 0% in week 3, 12.5% in week 4 (2/16), and 0% in week 5. Deaths were more frequent in patients >20 years of age (12/34; 35%) than in younger patients (1/27; 4%, p<0.01), and deaths were highest in 20- to 29-year-old patients (8/17; 47%). These findings suggest that the adults were affected earlier than children and teenagers. Two (4%) of the 50 patients treated with chloramphenicol died, whereas all 11 (100%) untreated patients died.

Nine of the 61 patients underwent some sort of CSF analysis. One had a positive direct microscopic examination. Four of the eight CSF samples taken in the field tested positive for meningococcus A in the rapid agglutination test; from one of these samples, serogroup A *N. meningitidis* was isolated. Thus, we considered that the epidemic was due to meningococcus A. The strain isolated was susceptible to major antibiotics, resistant to trimethoprim/sulfamethoxazole, and belonged to the epidemic clone A:4:P1.9 (sequence type ST-7), which was circulating simultaneously in north Cameroon and south Chad. The third pandemic caused by this strain began in China in 1993, causing large epidemics in Mongolia in 1994 and Moscow in 1996 (*4*). This sequence type seemed to emerge in Africa since 1995 (*5*), and researchers hypothesized that severe epidemics attributable to this ST-7 clone occurred in Cameroon and Niger, since such strains were circulating in the population and this ST-7 clone was responsible for severe outbreaks in Chad (1998) and Sudan (1999) (*5*). After meningococcus A was identified on March 22, the decision was made to vaccinate the local population. Polysaccharide A-C meningococcal vaccines were administered soon after week 6 in the outbreak area (i.e., after the epidemic ended).

Bechati is located in an area of tropical rainforest, with mountains at an altitude of ~1,000 m and deep humid valleys in which the villages are situated. This ecosystem is very different from the dry Sahelian ecosystem of the African meningitis belt. Nonetheless, an outbreak of serogroup A meningococcal infections occurred in this zone, so we investigated the possible causes of this epidemic.

The introduction of the strain into this remote population, probably in February 2000, was almost certainly favored by intense commercial exchanges with surrounding populations during the coffee harvest period at the end of the dry season, when roads are more navigable than during the rainy season. The epidemic strain then spread in the nonimmune population, which had no cohort immune barrier. All age groups had similar attack rates, in contrast to epidemics within the meningitis belt, which essentially affect children; the death rate in the absence of appropriate treatment was 100%. We showed that in 1999 to 2000 in Yaoundé, a large city situated in the tropical rainforest at about 600 km south of the meningitis belt, *N. meningitidis* was isolated in 13.4% of cases of bacterial meningitis, and most of the strains isolated belonged to serogroup A (*3*). Serogroup A and W135 meningococcal meningitis increased in Yaoundé between 1995 and 2000, possibly attributable to increases in human exchanges between the northern provinces (situated within the meningitis belt) and the central and southern provinces (*6*).

Other trigger factors frequently considered responsible for epidemics within the African meningitis belt are drought and the “Harmattan” wind because all major epidemics start at the driest period of the dry season and stop with the first rains. The Harmattan wind rarely reaches South Cameroon. Precipitation has been recorded over a number of years at Fontem Missionary Hospital. From 1995 to 1999, yearly rainfall averaged 2,300–2,500 mm, with only 0–50 mm from November to March. In the past 5 years, an average of no more than two consecutive months have been without rain, whereas almost four consecutive months without rain (December to the end of March) occurred just before the epidemic.

Thus, this outbreak appeared to result from several factors: 1) a virulent serogroup A strain belonging to ST-7 that had been responsible for recent epidemics in surrounding countries and was circulating in Cameroon; 2) the expansion of this strain, favored by the absence of an immune barrier in the population and by commercial exchanges; and 3) an exceptionally dry season. Outbreaks of meningococcal disease are not strictly bound to certain ecologic conditions occurring within the meningitis belt but may break out elsewhere. Since the epidemic reported here, another meningococcus A epidemic (~200 cases) has occurred at a similar equatorial latitude, near Bamenda (approximately 100 km north of Fontem), in 2001 (J. Kamgno, pers. comm.).

Health authorities should be aware of the possibility of such epidemics, be ready to alert medical practitioners and the public abouto them as they occur, and ensure that patients receive proper treatment and vaccines in these zones.
